# Osteoimmune microenvironmental failure in osteonecrosis of the femoral head: a necrosis-inflammation-repair loop and immune-editing-inspired framework for hip preservation

**DOI:** 10.3389/fimmu.2026.1891308

**Published:** 2026-08-03

**Authors:** Haotian Zheng, Chen Li, Xi Xie, Chunli Yao, Jiaxin Ma, Wei Chen, Xi Gao

**Affiliations:** 1 Heilongjiang University of Chinese Medicine, Harbin, Heilongjiang, China; 2 The First Affiliated Hospital of Heilongjiang University of Chinese Medicine, Harbin, Heilongjiang, China; 3 Traditional Chinese Medical Hospital of Gansu Province, Lanzhou, Gansu, China

**Keywords:** angiogenesis, bone marrow niche, cell death, hip-preserving therapy, osteoimmunology, osteonecrosis of the femoral head

## Abstract

Osteonecrosis of the femoral head (ONFH) is traditionally framed as structural failure after interrupted blood supply, yet this linear model does not explain why patients with similar radiographic stage may show markedly different pain trajectories, collapse kinetics, and hip-preservation outcomes. We propose that ONFH is better interpreted as an osteoimmune microenvironmental failure sustained by a self-reinforcing loop: ongoing cell-death input, maladaptive inflammatory reprogramming, bone-marrow niche dysfunction, and mechanical destabilization. In this framework, early sterile inflammation may support debris clearance, but persistent danger signaling can shift the lesion border into chronic, repair-incompetent inflammation. The marrow niche then transitions from a regenerative interface to a maladaptive state characterized by impaired stromal osteogenesis, endothelial dysfunction, uncoupled remodeling, and reduced load tolerance. To translate this model clinically, we organize intervention into three coordinated axes: entry blockade, niche reprogramming, and reconstruction. We further propose a dynamic decision path integrating stage, risk-factor burden, and niche activity, with serial imaging, biomarker trajectories, and functional outcomes guiding escalation.

## 1 Introduction

Osteonecrosis of the femoral head (ONFH) is a disabling disorder that often affects young and middle-aged adults, compromises joint-preserving options, and frequently culminates in femoral head collapse and total hip arthroplasty when diagnosis or biological containment is delayed. Current clinical management relies heavily on radiographic staging, lesion size, lesion location, and the presence or absence of subchondral collapse. These variables are indispensable for prognosis, but they remain incomplete descriptors of disease activity: patients with similar Association Research Circulation Osseous (ARCO), Ficat, or Japanese Investigation Committee stages may show divergent pain trajectories, repair responses, and collapse kinetics. Contemporary guidelines and consensus statements therefore increasingly emphasize early detection, risk-factor management, and the need to match intervention intensity to individual progression risk rather than to stage alone (1–7).

Epidemiological and imaging studies also show that ONFH is not a rare, uniform endpoint but a heterogeneous syndrome shaped by region, age, steroid exposure, alcohol use, systemic disease, coagulation status, and local biomechanical context. Large Chinese cohorts have highlighted the scale of nontraumatic ONFH and the importance of etiology-stratified analysis, while diagnostic guidelines and appropriateness criteria stress magnetic resonance imaging (MRI) for early disease and standardized reporting of lesion topography (8–12). Classification systems help communicate structural severity, but interobserver variability and the imperfect translation of static imaging into clinical outcome indicate that a deeper biological layer is missing from routine decision-making (13–15).

**Figure 1:**
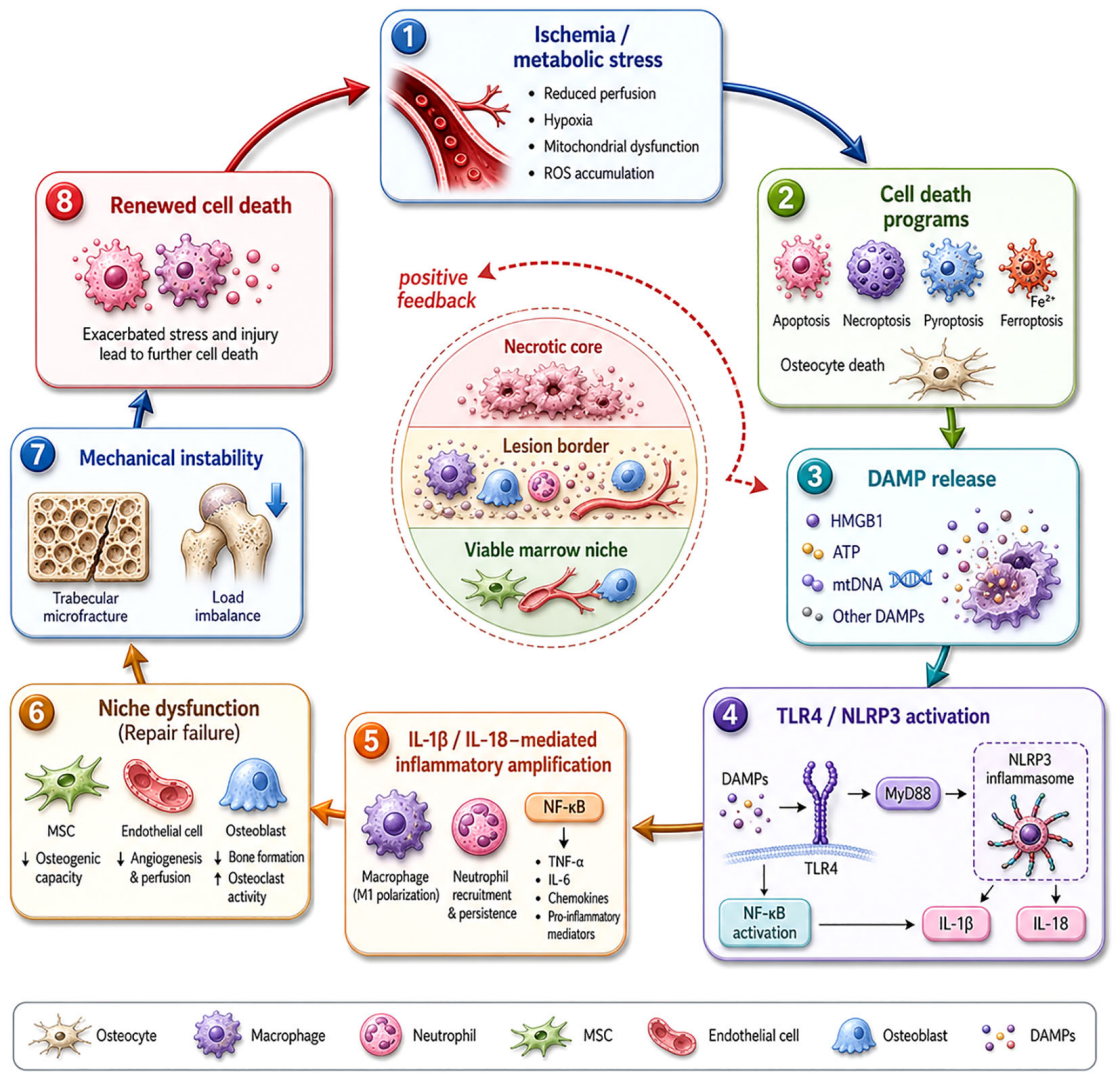
Proposed necrosis-inflammation-repair failure loop in ONFH. Ischemic, metabolic, thrombotic, and mechanical stress initiate multiple cell-death programs. DAMP release activates PRR and inflammasome signaling, shifting the marrow niche toward chronic inflammation, impaired osteogenesis, endothelial failure, uncoupled remodeling, and structural weakening. Mechanical microdamage then generates renewed cell death, closing the loop.

The missing layer is the activity state of the lesion border and adjacent marrow niche. Lesion size and position predict collapse because they define the residual load-bearing architecture, but they also approximate the scale of biologically active necrotic tissue, inflammation, vascular insufficiency, and reparative failure. Three-dimensional MRI quantification, preserved-angle concepts, volumetric assessment, and multidisciplinary diagnostic work increasingly show that the clinically important question is not simply whether necrosis is present, but whether the necrotic interface is being cleared, contained, repaired, or amplified into collapse (16–20). This review reframes ONFH as a dynamic osteoimmune disease in which cell death continuously feeds inflammatory circuits, inflammatory circuits reshape the bone marrow niche, and the remodeled niche weakens structural tolerance. We call this a necrosis-inflammation-repair failure loop and propose immune editing as a useful conceptual bridge between mechanism and individualized intervention (**Figure 1**).

The osteoimmune interpretation does not replace vascular, lipid, thrombotic, or mechanical models; it integrates them. Each classical model describes an upstream route through which the femoral head becomes vulnerable, whereas the immune-niche model explains why vulnerability can become self-maintaining after the first injury. The femoral head is a closed, load-bearing, highly specialized marrow environment in which a small change in perfusion, marrow pressure, trabecular integrity, or stromal-cell function may have a disproportionate effect on tissue survival. This anatomical constraint means that necrotic debris, hypoxic metabolites, oxidized lipids, and inflammatory mediators may accumulate at the border between dead and viable tissue, precisely where repair must occur. A biologically active border is therefore both a target and a threat: it contains the cells needed for regeneration, but it is also the site where continuing danger signals may reprogram those cells away from repair.

For immunology-focused readers, ONFH is an instructive example of sterile inflammation in a mechanically active tissue. Unlike inflammatory arthritis where adaptive autoimmunity often dominates, ONFH is primarily driven by endogenous DAMP-triggered sterile inflammation from necrotic cells. Unlike infection, the inciting signals are endogenous; unlike many autoimmune diseases, the main problem is not necessarily antigen specificity but failure to terminate danger signaling and restore a functional niche. The disease therefore sits at the intersection of regulated cell death, innate immune sensing, stromal-cell fate, angiogenesis, osteoclast-osteoblast coupling, and tissue biomechanics. This intersection justifies studying ONFH as a model of osteoimmune repair failure and provides a rational basis for combining immunomodulatory, regenerative, and structural treatments rather than evaluating each as an isolated intervention. In this review, we (1) propose a necrosis-inflammation-repair failure loop with three immune phases; (2) integrate upstream inputs and cell-death programs into this loop; (3) outline a three-axis intervention model (entry blockade, niche reprogramming, reconstruction); (4) provide a stage-risk-niche decision matrix; and (5) identify critical evidence gaps and future directions.

## 2 Literature scope and conceptual approach

### 2.1 Literature scope and method transparency

This is a narrative, hypothesis-generating review. We searched PubMed/MEDLINE, Web of Science Core Collection, Embase, and Cochrane Library for literature published from January 2000 to March 2026, with focused updates for 2024-2026. Search terms combined ONFH-related concepts (“osteonecrosis of the femoral head”, “avascular necrosis”, “nontraumatic osteonecrosis”) with mechanism and intervention terms (“cell death”, “necroptosis”, “pyroptosis”, “ferroptosis”, “DAMP”, “inflammasome”, “osteoimmunology”, “angiogenesis”, “core decompression”, “stem cell”, “scaffold”, “arthroplasty”). Priority was given to guidelines/consensus documents, mechanistic studies, translational investigations, prospective cohorts, and interventional studies. Because of heterogeneity across designs and outcomes, this manuscript does not perform pooled quantitative synthesis; instead, it provides structured mechanistic integration and clinically testable hypotheses.

The literature was organized around five intersecting domains: clinical staging and prognosis; ischemic, metabolic, and thrombotic risk inputs; regulated and non-regulated cell-death programs; DAMP-pattern recognition receptor (PRR) signaling and osteoimmunology; and hip-preserving or reconstructive interventions. Priority was given to peer-reviewed guidelines, consensus documents, mechanistic studies, translational models, clinical cohorts, and recent reviews. The purpose was not to provide a comprehensive catalogue of every ONFH mechanism, but to build a coherent disease model that can be tested clinically and experimentally.

The central premise is that ONFH progression is governed by an imbalance between three rates: the rate at which necrotic and stressed cells continue to release inflammatory and oxidative signals, the rate at which immune and stromal cells can clear and resolve those signals, and the rate at which the osteovascular niche can rebuild mechanically competent bone. When the first rate exceeds the latter two, ONFH behaves as an expanding microenvironmental failure. When clearance and repair catch up, the lesion may stabilize even when imaging reveals residual necrotic architecture. This framing is compatible with classic vascular and mechanical theories, but it adds an explicit immunological and temporal dimension that explains why single-target interventions often produce inconsistent results.

## 3 Upstream inputs: ischemia, steroid exposure, lipid stress, coagulation, and local overload

The initial insult in nontraumatic ONFH is commonly described as a vascular event, but the mechanisms leading to impaired femoral head perfusion are diverse. Glucocorticoids can induce adipocyte hypertrophy, marrow pressure elevation, endothelial dysfunction, osteoblast and osteocyte apoptosis, oxidative injury, and impaired HIF-1alpha/VEGF signaling. Alcohol and lipid disorders can reinforce adipogenic expansion and lipotoxic stress. Coagulation abnormalities and hypofibrinolysis may contribute to microthrombotic obstruction, whereas vasculitis-like injury, autoimmune disease, and systemic inflammation may reduce the resilience of the femoral head microcirculation. These pathways do not merely cause a one-time ischemic episode; they can remain active after diagnosis and continue to drive local tissue stress (21–30).

A key clinical implication is that the etiologic label should be considered an ongoing input, not simply a historical cause. A patient with continued high-dose glucocorticoid exposure, uncontrolled alcohol intake, persistent dyslipidemia, or active inflammatory disease continues to deliver stress to the lesion even after a core decompression or biological adjunct. Conversely, an early-stage lesion in a patient whose systemic inputs are rapidly reduced may behave differently despite a similar MRI stage. This distinction supports a dynamic risk-factor burden score integrated with structural stage and microenvironmental readouts. Etiology-specific differences in upstream stressors, niche signatures, inflammatory profiles, and clinical implications are summarized in **Table 1**.

**Table 1:** Comparison of microenvironmental features across ONFH etiologies.

Etiology	Dominant upstream stressors	Niche signature	Inflammation profile	Clinical implication
Steroid-induced ONFH	Glucocorticoid exposure, adipogenic shift, endothelial dysfunction	High adipogenic drift, impaired osteogenesis, reduced angiogenic coupling	Persistent sterile inflammation with oxidative stress vulnerability	Early risk-factor control plus niche reprogramming is critical
Alcohol-associated ONFH	Lipid dysregulation, oxidative injury, metabolic stress	Marrow lipid expansion, trabecular fragility, delayed vascular recovery	Chronic low-grade inflammatory activation with remodeling imbalance	Emphasize metabolic correction and load management
Traumatic ONFH	Acute vascular interruption and mechanical disruption	Localized ischemic core with border-zone reparative pressure	Inflammation often phase-constrained but can persist with microdamage	Prioritize structural timing with biologic support in transition phase
Idiopathic ONFH	Mixed latent risk inputs, possible microvascular/coagulation contribution	Heterogeneous niche activity and progression trajectories	Variable immune activity requiring dynamic stratification	Use stage-risk-niche monitoring for individualized escalation

Steroid-associated ONFH is a particularly useful lens because glucocorticoids affect nearly every compartment implicated in the loop. They can suppress osteoblastogenesis, promote adipogenic differentiation of marrow stromal cells, alter lipid deposition, induce endothelial dysfunction, influence coagulation and fibrinolysis, and increase susceptibility to oxidative injury. In the short term, glucocorticoids may suppress systemic inflammation, but in the femoral head they can create a paradoxical microenvironment in which stromal repair is weakened, lipid stress is increased, and dying cells provide new inflammatory inputs. This may explain why steroid-induced ONFH often progresses despite the anti-inflammatory properties of the drug class.

Mechanical input deserves equal attention. The superior-lateral femoral head is exposed to high load, and lesion location strongly affects the probability that damaged trabeculae will fail. Mechanical overload does not simply fracture weakened bone; it also changes cell behavior. Microcracks, matrix deformation, and altered interstitial fluid flow can injure osteocytes, activate osteoclasts, and generate matrix-derived DAMPs. As a result, the mechanical environment can convert a contained biological lesion into a structural catastrophe and then feed back to the immune system through additional cell death and matrix degradation.

## 4 Cell-death programs as the persistent entry point of the loop

Cell death is the first biological language spoken by an ischemic femoral head. Classical apoptosis, secondary necrosis after failed efferocytosis, regulated necrosis, pyroptosis, and ferroptosis are not mutually exclusive in ONFH. They represent different ways in which cells die, communicate danger, and influence surrounding immune and stromal cells. Modern nomenclature emphasizes that regulated cell death is defined by molecular machinery and that several death pathways can convert from relatively silent cell loss into highly immunogenic tissue damage when clearance is delayed or membrane integrity is lost (31–34).

Apoptosis is often considered immunologically quiet because intact apoptotic bodies can be removed by macrophages and neighboring cells. In ischemic bone, however, low perfusion, limited immune access, and excessive cell burden can overwhelm efferocytosis. Apoptotic osteocytes, osteoblasts, endothelial cells, and marrow stromal cells may then progress to secondary necrosis, creating a delayed danger signal. This process helps explain why a femoral head lesion can remain biologically active after the initial vascular interruption. Histological and clinical studies demonstrate extensive osteocyte apoptosis in ONFH and glucocorticoid-induced hip osteonecrosis, while experimental work links oxidative stress, diminished vascularity, and decreased bone strength to early osteocyte and endothelial injury (35–40).

Necroptosis is a regulated, lytic form of cell death involving receptor-interacting protein kinases and MLKL-dependent membrane disruption. Its relevance to ONFH is conceptual as well as mechanistic: necroptosis directly couples cell death to DAMP release and sterile inflammation. When necroptosis occurs in mechanically stressed bone or hypoxic marrow, it can convert local injury into a macrophage-activating signal. Although direct ONFH-specific evidence remains incomplete, the necroptosis literature provides a plausible pathway by which cytokine-rich or TNF-associated microenvironments amplify death-induced inflammation rather than resolving it (41–45).

Pyroptosis adds another layer because it is both a death program and an inflammatory cytokine-release platform. Inflammasome activation leads to caspase-mediated gasdermin cleavage, membrane pore formation, and release of interleukin-1beta and interleukin-18. In ONFH, pyroptosis-related biomarkers and inflammasome-linked mechanisms have been implicated in steroid-induced disease, and recent translational studies suggest that the pyroptosis axis can influence both MSC dysfunction and endothelial injury. The practical consequence is that a lesion dominated by inflammasome activity may require not only structural decompression but also inflammatory tone reduction and niche reprogramming (46–51).

Ferroptosis is especially attractive in ONFH because it connects three recurrent features of the disease: iron-dependent lipid peroxidation, oxidative stress, and lipid-metabolic disturbance. Glucocorticoids can alter lipid handling and redox balance, while hypoxia-reoxygenation and mitochondrial stress may increase peroxidized phospholipids. Experimental studies implicate the P53/SLC7A11/GPX4 pathway in steroid-induced ONFH, supporting the idea that ferroptosis can provide continuous oxidative and DAMP input in patients with persistent metabolic or steroid-related stress (44, 52–56).

These death programs should not be viewed as separate diagnostic silos. In a pre-collapse lesion, apoptosis may dominate in osteocytes and endothelial cells, ferroptosis may emerge in lipid-stressed marrow elements, pyroptosis may appear in macrophages or injured stromal cells, and necroptosis may intensify when cytokine and mechanical injury converge. The stage-specific dominance of a pathway may determine whether anti-oxidative, anti-inflammatory, pro-angiogenic, or structural strategies are most rational. The unresolved challenge is to develop clinically feasible biomarkers that can infer the death-program mix without requiring destructive tissue sampling.

A practical way to interpret these death programs is to ask three questions: how much membrane rupture occurs, which inflammatory mediators are released, and whether nearby phagocytes can clear the residue before secondary damage accumulates. Apoptosis with efficient efferocytosis may support resolution, whereas pyroptosis, necroptosis, and secondary necrosis tend to expose intracellular material directly to PRRs. Ferroptosis may be less visibly lytic at first, but lipid peroxidation products can propagate oxidative stress and alter macrophage and stromal-cell behavior. Thus, the inflammatory value of cell death depends not only on the death pathway but also on the capacity of the local niche to buffer its products.

The temporal sequence is likely non-linear. To better understand the temporal interaction of regulated cell death programs, ONFH can be viewed as a stage-dependent process. In early pre-collapse lesions, apoptosis and secondary necrosis may dominate in osteocytes and endothelial cells, generating initial DAMP signals for immune recruitment. During transitional lesions, pyroptosis, ferroptosis, and necroptosis may become increasingly prominent under persistent inflammatory, oxidative, and lipid stress. In chronic escape lesions, sustained necrotic signaling may maintain inflammatory amplification, impair osteogenic regeneration, and promote remodeling failure. This temporal recycling is why a lesion may remain active even when the original vascular insult is no longer measurable **Table 2**.

**Table 2:** Stage-dependent cell death programs and immune consequences in ONFH.

Stage	Dominant death pathway	Major cells	Signals	Outcome
Early pre-collapse	Apoptosis/secondary necrosis	Osteocytes/endothelial cells	HMGB1, mtDNA	Debris clearance
Transition	Pyroptosis/ferroptosis/necroptosis	Macrophages/MSCs	IL-1β, IL-18, oxidized lipids	Inflammation amplification
Chronic escape	Persistent necrotic signaling	Multiple marrow cells	ATP, DAMPs	Repair failure

## 5 DAMP-PRR signaling and an immune-editing-like framework

Dying and necrotic cells release DAMPs such as HMGB1, mitochondrial DNA, ATP, heat-shock proteins, oxidized lipids, and extracellular matrix fragments. These molecules engage PRRs including Toll-like receptors, NOD-like receptors, and inflammasome components, thereby transforming local tissue damage into innate immune activation. The danger model and the sterile inflammation literature show that such signaling is not inherently pathological; it is necessary for debris clearance and repair initiation. Pathology emerges when the signal is excessive, repetitive, or no longer synchronized with the reparative capacity of the tissue (57–68).

Necrotic bone itself can behave as an immunostimulatory substrate. Experimental work demonstrates that necrotic bone can stimulate macrophage pro-inflammatory responses through TLR4, and DAMPs extracted from necrotic femoral head tissue can inhibit osteogenic differentiation while promoting fibrogenic behavior in MSCs. These findings provide a mechanistic bridge between cell death and repair failure: the material that must be repaired also generates signals that impair the cells responsible for repair (69, 70). Inflammation in ONFH therefore should not be reduced to the presence of cytokines; it is a spatiotemporal program in which necrotic matrix, immune cells, stromal cells, and vascular cells continually instruct each other.

We propose an immune-editing-like model with three phases. This analogy is used strictly to describe temporal phases of immune-tissue interaction, not to imply neoplastic transformation or tumor-like immune evasion. Importantly, this immune-editing analogy is conceptual rather than oncological. Unlike cancer immune editing, ONFH does not involve malignant transformation, tumor-associated antigens, clonal expansion of abnormal cells, or immune escape from antitumor surveillance. The three proposed phases describe temporal changes in tissue injury, innate immune activation, and repair competence. Therefore, “chronic escape” refers to escape from effective tissue repair control rather than neoplastic immune escape. In the early containment phase, DAMP-PRR signaling recruits and activates innate immune cells, promotes debris clearance, and may transiently support vascular and osteogenic repair. In the transitional coexistence phase, necrotic input, inflammation, angiogenesis, and bone formation coexist at the lesion border; this is the most important therapeutic window because small changes in risk inputs or niche support can tip the system toward stabilization or progression. In the chronic escape phase, persistent hypoxia, lipid toxicity, endothelial failure, and abnormal loading establish an autonomous inflammatory state. At that point inflammation maintains itself, suppresses osteogenesis, uncouples remodeling, and accelerates structural failure.

This analogy is not meant to imply that ONFH is cancer. Rather, it borrows the temporal logic of immune editing: an early immune reaction can be protective, a middle phase can contain but not eliminate the lesion, and a late phase can lose control and permit disease escape. The model also helps reconcile apparently conflicting therapeutic observations. Complete suppression of early inflammation could impair clearance; delayed suppression of entrenched inflammation could be insufficient; and biological reconstruction without controlling active danger input may fail because the implanted or recruited cells enter a hostile niche.

The immune-editing analogy is used here as a heuristic, not as a claim that ONFH is a neoplasm. In cancer, immune editing describes elimination, equilibrium, and escape. In ONFH, an early containment phase corresponds to early debris clearance and recruitment of reparative programs; a transitional coexistence phase corresponds to partial containment in which inflammation and repair coexist; and a chronic escape phase corresponds to chronic danger signaling, niche exhaustion, and structural progression. The value of the analogy is temporal: it reminds clinicians that the same immune process can be beneficial, ambiguous, or harmful depending on when and where it occurs.

In the early containment phase, macrophages and other innate immune cells should be permitted to clear dead cells and matrix fragments, provided that the intensity is proportionate and transient. In the transitional coexistence phase, the border zone becomes the key therapeutic window because the femoral head is not yet structurally lost, but repair has not fully gained dominance. In the chronic escape phase, immune signaling has become uncoupled from effective regeneration; inflammatory mediators, oxidative stress, endothelial injury, and mechanical microfracture maintain each other. Identifying which phase a patient occupies is more clinically meaningful than asking whether inflammation is simply present or absent.

## 6 Bone marrow niche remodeling: from regenerative interface to maladaptive ecosystem

The bone marrow niche in osteonecrosis of the femoral head (ONFH) is not a passive container for necrotic tissue but a dynamic multicellular ecosystem that determines whether tissue injury is contained or progresses toward structural collapse. This niche integrates osteocytes, macrophages, mesenchymal stromal cells (MSCs), endothelial cells, osteoblasts, osteoclasts, marrow adipocytes, extracellular matrix components, and mechanical signals. Under physiological conditions, these components coordinate immune surveillance, angiogenesis, osteogenesis, and bone remodeling. In ONFH, persistent ischemic, metabolic, oxidative, and mechanical stresses disrupt this coordination, transforming a regenerative interface into a maladaptive microenvironment (71–81).

### 6.1 Osteocytes: initiating danger signals and mechanical sensing

Osteocytes represent one of the earliest cellular targets of ischemic, glucocorticoid-associated, and oxidative stressin ONFH. Although traditionally regarded as terminally differentiated bone cells, osteocytes actively regulate mechanical adaptation, bone remodeling, and communication between skeletal and immune compartments. Excessive oxidative stress, impaired perfusion, and metabolic disturbance induce osteocyte apoptosis and secondary necrosis, generating danger-associated molecular patterns (DAMPs), including high mobility group box 1 (HMGB1) and mitochondrial DNA.

The loss of osteocytes is therefore not merely a consequence of tissue injury but an active mechanism that initiates inflammatory amplification and mechanical instability. Osteocyte-derived danger signals can recruit and activate immune cells, while impaired osteocyte mechanosensing reduces the ability of bone tissue to adapt to mechanical loading, accelerating microdamage accumulation and structural deterioration (35–39, 82).

### 6.2 Macrophages: from debris clearance to chronic inflammatory persistence

Macrophages are central regulators of the lesion border in ONFH. During early disease stages, macrophages contribute to tissue recovery through efferocytosis, clearance of necrotic debris, secretion of pro-resolving mediators, and support of angiogenic responses. This reparative function is essential for transforming cell death signals into effective tissue remodeling.

However, persistent exposure to DAMPs, hypoxia, oxidized lipids, and inflammatory cytokines can reprogram macrophages toward chronic inflammatory states. Activation of pathways including Toll-like receptor 4 (TLR4)/NF-κB and inflammasome signaling promotes sustained inflammatory mediator production and may enhance osteoclastogenic activity. Therefore, macrophage function in ONFH should not be interpreted through a simple M1/M2 classification but rather according to whether macrophages maintain effective debris clearance, angiogenesis, matrix repair, and balanced remodeling (72–76).

### 6.3 Mesenchymal stromal cells: osteogenic-adipogenic fate imbalance

MSCs and skeletal progenitor cells represent the primary regenerative population responsible for osteogenic reconstruction after injury. However, ONFH-associated niche dysfunction frequently converts MSCs from reparative cells into dysfunctional populations with impaired regenerative capacity.

Glucocorticoid exposure, oxidative stress, inflammatory cytokines, HMGB1-rich DAMP environments, and extracellular matrix degradation suppress osteogenic differentiation, promote adipogenic lineage commitment, reduce migration capacity, and induce senescence-like phenotypes. Thus, the major problem is not simply insufficient MSC numbers but impaired MSC functional competence and abnormal lineage allocation.

The imbalance between osteogenesis and adipogenesis represents a central mechanism linking marrow niche dysfunction with structural deterioration. Restoration of MSC-mediated repair therefore requires not only cell replacement strategies but also correction of inflammatory, metabolic, and mechanical abnormalities within the local niche (70, 83–88).

### 6.4 Endothelial cells and type H vessels: failure of angiogenic-osteogenic coupling

Bone regeneration requires coordinated communication between vascular and skeletal systems. Endothelial cells, particularly specialized Type H vessels, provide essential signals that regulate osteoprogenitor activity and bone formation. VEGF signaling and endothelial Notch pathways represent key mechanisms maintaining angiogenic-osteogenic coupling.

In ONFH, endothelial injury caused by hypoxia, oxidative stress, glucocorticoids, and metabolic disturbance reduces vascular recovery and nutrient delivery. Impaired HIF-1α/VEGF signaling further limits angiogenic responses and compromises osteogenic activity. As vascular support declines, the marrow environment becomes hypoxic and metabolically restricted, allowing inflammatory signaling and cell death programs to persist.

Therefore, endothelial dysfunction represents not only vascular insufficiency but also failure of the regenerative communication network between blood vessels and bone-forming cells (89–94).

### 6.5 Osteoblast–osteoclast remodeling axis: uncoupled bone turnover

Normal bone repair requires coordinated interaction between osteoblast-mediated bone formation and osteoclast-mediated bone resorption. Osteoimmunological regulation through pathways such as receptor activator of nuclear factor-κB ligand (RANKL)/osteoprotegerin (OPG) maintains this balance.

In ONFH, persistent inflammatory signaling and altered niche conditions disrupt this coupling. Enhanced osteoclast activation may accelerate resorption around necrotic regions, whereas osteoblast differentiation and matrix production remain insufficient. This imbalance results in progressive trabecular weakening and reduced mechanical competence of the femoral head.

Thus, structural collapse is not simply caused by the presence of necrotic bone but reflects failure of coordinated remodeling between bone resorption and regeneration (78–81).

### 6.6 Marrow adipocytes: lipotoxicity and intramedullary pressure elevation

Marrow adipocytes represent an important metabolic component of niche dysfunction, particularly in steroid-associated ONFH. Excessive adipogenic differentiation of MSCs increases marrow lipid accumulation and alters the metabolic environment of the femoral head.

Expanded marrow adipocytes contribute to disease progression through multiple mechanisms. First, lipid accumulation promotes lipotoxic stress and oxidative injury. Second, increased lipid peroxidation enhances susceptibility to ferroptotic cell death. Third, adipocyte hypertrophy increases intramedullary pressure, reduces vascular space, and further compromises local perfusion.

Therefore, marrow adipocytes function as active regulators of metabolic stress and vascular dysfunction rather than passive storage cells (22–24, 86–88).

### 6.7 Extracellular matrix and mechanical feedback: transition from niche failure to collapse

The extracellular matrix and mechanical environment ultimately determine whether biological dysfunction progresses toward structural failure. Matrix degradation, altered collagen organization, fibrosis, and microcrack accumulation disrupt load distribution within the femoral head.

Mechanical overload subsequently induces additional osteocyte and endothelial injury, generating new DAMP release and reinforcing inflammatory activation. Thus, the relationship between niche dysfunction and mechanical collapse is bidirectional: cell death promotes inflammation, inflammation disrupts the niche, niche failure weakens structural integrity, and mechanical damage generates further cellular injury.

This feedback mechanism explains why ONFH may continue progressing even after the initial ischemic insult has resolved and highlights the importance of integrating biological activity with structural assessment (16–20, 69, 70).

Finally, the extracellular matrix and mechanical environment transform biological failure into collapse. Matrix degradation, fibrogenic stromal behavior, disorganized collagen, and microcrack accumulation alter local stress transfer. Mechanical overload then injures additional osteocytes and endothelial cells, generating new death signals and DAMP release. The result is a feedback loop: death induces inflammation, inflammation distorts the niche, niche failure weakens structure, weakened structure causes microdamage, and microdamage induces more death.

## 7 The necrosis-inflammation-repair failure loop

The integrated ONFH loop can be summarized as follows: ischemic or metabolic stress initiates multiple death programs; cell death releases DAMPs and oxidized molecular patterns; PRR and inflammasome signaling activate innate immune cells; immune activation initially supports clearance but later becomes chronic; chronic inflammation impairs MSC osteogenesis, endothelial repair, and remodeling balance; impaired repair reduces structural competence; mechanical stress and microfracture generate new death input. This loop explains why ONFH is often progressive even when the initiating exposure has ended, and why some lesions collapse rapidly before large changes are visible on plain radiographs. The major mechanistic modules in this loop and their therapeutic implications are summarized in **Table 3**.

**Table 3:** Mechanistic modules in the necrosis-inflammation-repair failure loop.

Module	Dominant signals	Microenvironmental consequence	Therapeutic implication
Cell-death input	Apoptosis, secondary necrosis, necroptosis, pyroptosis, ferroptosis	DAMP release, oxidative burden, inflammatory cytokines	Reduce steroid/metabolic stress; target oxidative, inflammasome, or ferroptotic pathways
Innate immune activation	TLR4/NF-kappaB, NLRP3, HMGB1, mitochondrial DAMPs	Debris clearance early; chronic inflammation if input persists	Time immunomodulation to disease phase rather than suppress all inflammation
MSC and stromal mismatch	Low osteogenesis, adipogenic drift, senescence-like or fibrogenic behavior	Poor matrix formation and weak repair interface	Improve niche quality before or during cell/scaffold therapy
Endothelial failure	Reduced HIF-1alpha/VEGF signaling, microvascular injury	Hypoxia, poor nutrient delivery, low angiogenic-osteogenic coupling	Combine decompression, endothelial protection, angiogenic support
Remodeling and mechanics	RANKL/OPG imbalance, microcracks, matrix disorganization	Subchondral weakening and collapse	Escalate to structural reconstruction when load tolerance fails

Three clinical phenotypes can be derived from this model. A low-activity contained phenotype has residual necrotic architecture but declining death and inflammatory signals, improving function, and stable imaging. A transition phenotype has preserved gross structure but high biological activity, pain flares, rising marrow edema, or early subchondral stress; this phenotype may benefit most from combined biological and mechanical interventions. A chronic escape phenotype has entrenched inflammation, marked vascular failure, progressive microfracture, and impaired load tolerance; in this phenotype structural reconstruction or joint replacement may become more appropriate than repeated low-intensity biological therapy.

The model also encourages separation of therapeutic success into three endpoints. Biological success means reducing death input and inflammatory tone. Niche success means restoring osteogenic and angiogenic competence. Structural success means preserving spherical femoral head integrity and load-bearing function. A treatment may achieve one endpoint but not another. For example, core decompression can reduce intraosseous pressure and improve perfusion but may not correct systemic steroid input. MSC delivery can add reparative cells but may fail in a DAMP-rich, hypoxic, inflammatory niche. Anti-inflammatory treatment can reduce pain but may not restore mechanical strength. Multidimensional endpoints are therefore necessary.

## 8 Three-axis intervention framework

The first axis is entry blockade. Its goal is to reduce the continuous input of necrotic, metabolic, oxidative, and mechanical stress. Clinically, this includes minimizing glucocorticoid exposure when medically feasible, controlling alcohol intake, treating dyslipidemia and metabolic syndrome, addressing coagulation or autoimmune contributors, and applying stage-appropriate load modification. Early MRI-based lesion quantification can identify patients whose structural risk is high despite limited symptoms, while functional assessment can reveal those whose microenvironment may already be active. Entry blockade is not a passive conservative strategy; it is the active removal of inputs that feed the loop.

The second axis is niche reprogramming. This axis aims to convert a chronic pro-inflammatory, hypovascular, adipogenic niche into a repair-supporting niche. Potential strategies include modulation of macrophage and neutrophil programs, inhibition of excessive inflammasome or NF-kappaB signaling, reduction of oxidative and ferroptotic stress, protection of endothelial cells, promotion of type H vessel-like angiogenesis, and restoration of osteoblast-osteoclast coupling. Preclinical and early translational studies involving extracellular vesicles, miRNA-modified stem cells, endothelial protection, autophagy modulation, and pyroptosis-related targets illustrate the breadth of possible approaches (95–105).

The third axis is structural reconstruction. Core decompression, multiple drilling, bone grafting, vascularized fibular grafting, tantalum rods, porous scaffolds, biologically active materials, osteotomy, and eventual arthroplasty all target the mechanical consequences of the loop. These strategies should not be understood as separate from immunology. Decompression changes perfusion and pressure; scaffolds instruct immune and stromal cells; grafts alter matrix architecture; and arthroplasty removes a chronically inflamed, mechanically incompetent tissue environment. Classic and modern studies of core decompression and cell-based augmentation support the principle that mechanical and biological strategies are most effective when used before irreversible collapse, but outcomes remain variable because microenvironmental activity is rarely measured at baseline (106–118).

**Figure 2:**
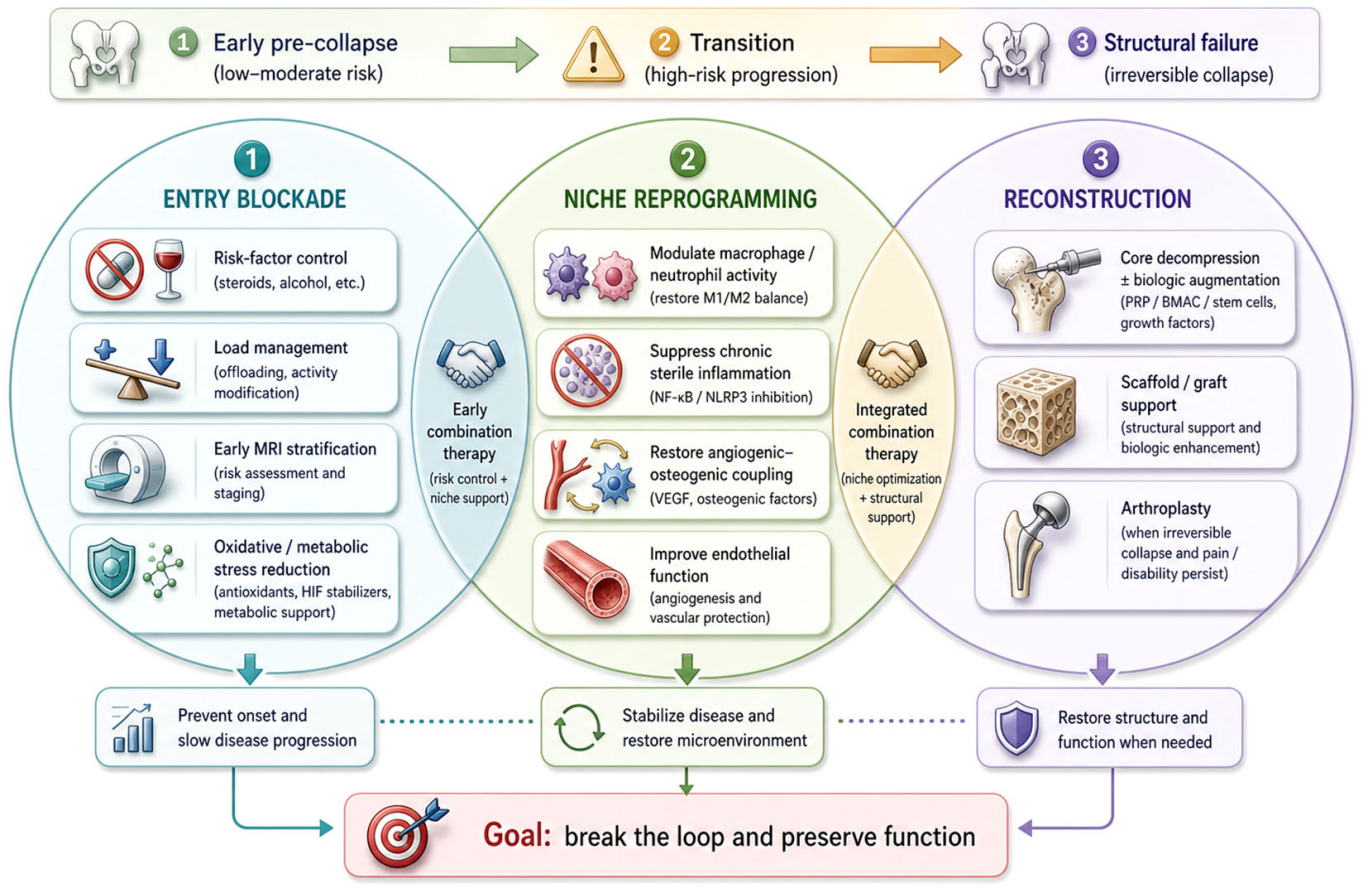
Three-axis intervention model. Entry blockade reduces ongoing necrotic, metabolic, oxidative, and mechanical inputs. Niche reprogramming restores immunovascular and osteogenic competence. Reconstruction preserves or replaces load-bearing architecture. The axes can be combined according to stage, risk-factor burden, and niche activity.

The three axes should be combined, not sequenced rigidly. A pre-collapse lesion with high steroid burden and marrow edema may require entry blockade, niche reprogramming, and decompression simultaneously. A small low-activity lesion may be monitored with risk-factor optimization and serial imaging. A large lateral lesion with early subchondral fracture may need rapid structural escalation even if systemic inflammation is controlled. The intervention intensity should be determined by the intersection of structural stage, risk-factor burden, and niche activity. Several clinically available or investigational interventions may influence specific components of the osteoimmune failure loop, although most remain limited by heterogeneous evidence and lack of biomarker-guided patient selection. Their mechanistic relevance within the proposed framework is summarized in **Table 4**. **Figure 2** illustrates how these axes can be combined according to stage, risk-factor burden, and niche activity.

**Table 4:** Clinical interventions and their mechanistic relevance within the osteoimmune framework of ONFH.

Intervention	Mechanistic relevance in ONFH	Current evidence and translational status
Bisphosphonates	Modulation of osteoclast-mediated bone resorption and preservation of trabecular structure	Clinical and observational studies; efficacy remains stage-dependent (107–118)
Statins	Regulation of lipid metabolism, reduction of oxidative stress, and endothelial protection	Observational and experimental evidence; prospective validation is required
Anticoagulation	Reduction of microvascular thrombosis and improvement of coagulation-related vascular stress	Selected clinical studies in patients with thrombophilic risk factors
Hyperbaric oxygen	Improvement of local hypoxia, enhancement of angiogenic responses, and reduction of oxidative stress	Clinical studies, mainly in early-stage disease; further biomarker-guided validation is required (106)
MSC/exosomes	Restoration of osteogenic capacity, angiogenic-osteogenic coupling, and marrow niche function	Preclinical and early translational studies; clinical standardization remains limited (95–104)

Entry blockade should be interpreted broadly. It includes reducing or tapering glucocorticoid exposure when medically possible, managing lipids and alcohol, addressing coagulation risk when appropriate, controlling systemic inflammatory disease, limiting high-impact loading during active disease, and optimizing comorbidities that impair endothelial function. In research settings, entry blockade may also include antioxidant, anti-ferroptotic, or endothelial-protective approaches. The goal is not merely risk-factor counseling; it is to reduce the ongoing biological input that prevents the lesion from exiting the inflammatory phase.

Niche reprogramming is the least mature axis clinically but the most important for immunology. Potential strategies include macrophage phenotype modulation, inflammasome pathway control, promotion of efferocytosis, endothelial repair, angiogenic-osteogenic coupling, restoration of MSC potency, and local delivery of instructive biomaterials or extracellular vesicles. These approaches should be tested with phase-specific endpoints. For example, in a transition lesion, a successful niche therapy might reduce marrow edema, normalize inflammatory markers, improve perfusion, and stabilize pain before any major structural change is visible.

Reconstruction should be timed to prevent mechanical failure rather than to rescue a biologically uncontrolled lesion after collapse. Core decompression may be most effective when it reduces intraosseous pressure and improves perfusion while the border zone remains capable of repair. Grafts, scaffolds, and vascularized constructs may be more successful when the local immune environment is not strongly in the chronic escape phase. Conversely, when collapse and cartilage damage have crossed an irreversible threshold, arthroplasty can be the most rational reconstruction because it removes a failed load-bearing system rather than attempting to biologically revive it.

## 9 Biomarkers, imaging windows, and dynamic interpretation

A central weakness in ONFH research is the absence of standardized, mechanism-linked activity markers. A useful biomarker framework should capture five domains: cell death and oxidative stress; inflammatory and immune activation; bone remodeling; endothelial and angiogenic function; and imaging-defined structural risk. Candidate markers include lipid peroxidation products, GPX4/SLC7A11-related signatures, HMGB1 or mitochondrial DAMPs, IL-1beta and inflammasome-related markers, RANKL/OPG balance, bone turnover markers, VEGF-related signals, endothelial injury markers, exosomal microRNAs, and MRI-derived lesion volume, marrow edema, subchondral fracture, and radiomic features. The candidate domains and practical sampling windows are organized in **Table 5**.

**Table 5:** Candidate biomarker domains and sampling windows.

Domain	Examples	Interpretation
Cell death/oxidative stress	Lipid peroxidation, GPX4/SLC7A11-related signals, HMGB1, mitochondrial DAMPs	Persistent necrotic or ferroptotic input
Inflammation	IL-1beta, IL-18, TNF-alpha, NLRP3-related signatures, neutrophil or macrophage phenotypes	Clearance, transition, or chronic escape activity
Bone remodeling	RANKL/OPG, CTX, P1NP, osteocalcin, alkaline phosphatase	Coupled repair versus resorptive imbalance
Vascular/endothelial	VEGF-related signals, endothelial injury markers, perfusion MRI	Angiogenic competence and oxygen recovery
Imaging and function	MRI volume, preserved angles, marrow edema, radiomics, pain, gait/load tolerance	Structural risk and clinical meaning of biological change
Windows	Baseline; 2–6 weeks; around 3 months; every 3–6 months in active disease	Use trajectories rather than isolated single values

Ferroptotic activity should not be inferred from a single marker. A combined assessment incorporating lipid peroxidation products (MDA, 4-HNE), antioxidant defense pathways (GPX4/SLC7A11), iron metabolism parameters, and molecular signatures may provide a more reliable biological interpretation.

Currently, niche activity should be regarded as a conceptual biological classification rather than a validated clinical score. No standardized cutoff values have been established for HMGB1, mitochondrial DNA, GPX4 activity, ferroptosis-related metabolites, or endothelial injury markers in ONFH populations. Prospective multicenter studies are required to define clinically meaningful thresholds.

**Figure 3:**
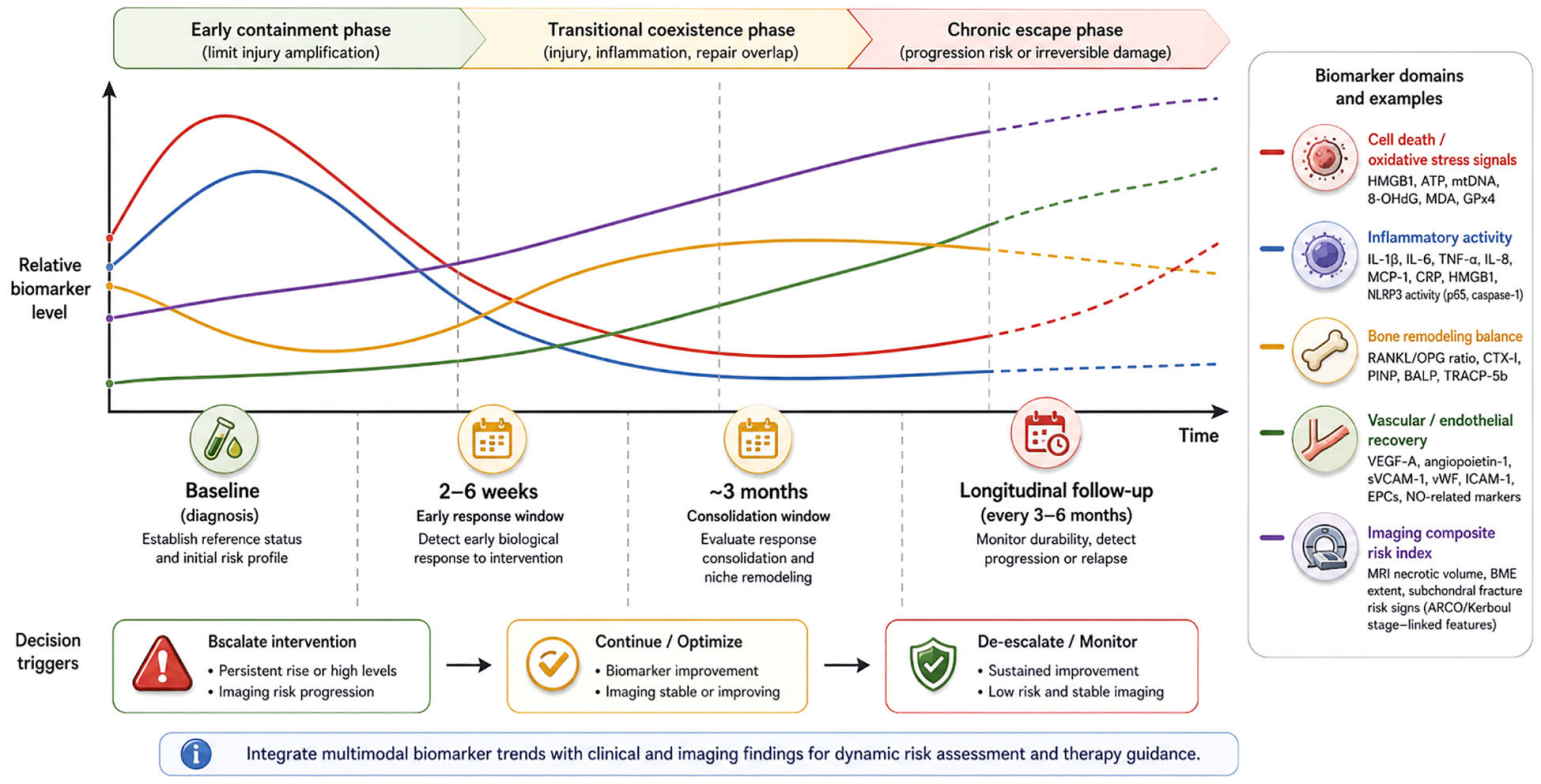
Candidate biomarker and sampling trajectory. Baseline assessment defines stage-risk-niche status. A 2- to 6-week window evaluates early biological response; an approximately 3-month window guides escalation; 3- to 6-month follow-up supports re-stratification. Imaging, biomarkers, and function should be interpreted together.

Single measurements are less informative than trajectories. Baseline testing can define the initial combination of stage, risk, and niche activity. A 2- to 6-week window can reveal whether entry blockade or anti-inflammatory strategies are reducing activity. A 3-month window can guide escalation toward decompression, biological augmentation, or intensified niche therapy. Thereafter, 3- to 6-month intervals may be appropriate for high-risk or biologically active lesions, whereas stable low-risk lesions may be followed less frequently. Pain, walking tolerance, analgesic requirement, and patient-reported function must be interpreted alongside imaging because biological improvement without functional benefit may not represent meaningful disease control. **Figure 3** summarizes this trajectory-based interpretation path.

Recent biomarker discovery studies support this trajectory-based approach. Serum WGCNA and machine-learning analyses, exosomal miR-135b-5p, pyroptosis-related signatures, IRF8-linked immune infiltration, endoplasmic reticulum stress genes, and MSC-pyroptosis modules all illustrate that circulating and computational biomarkers are feasible, although most require external validation and harmonized sampling (119–123). A future ONFH activity score should be clinically practical, reproducible across centers, and linked to predefined treatment decisions rather than serving as a descriptive research output alone.

A clinically useful biomarker panel should satisfy four conditions. It should be measurable with standardized protocols, change over a clinically meaningful time scale, map to a mechanism that can be treated, and improve decision-making beyond existing imaging. Many candidate molecules currently fail at least one of these conditions. For example, a cytokine may be biologically plausible but too variable for routine classification, while a radiomic feature may predict collapse but not reveal which intervention is best. Combining domains is therefore essential: imaging can define structural vulnerability, whereas blood or marrow-derived markers may define activity and therapeutic direction.

Sampling windows should also be aligned with intervention biology. A drug or risk-factor intervention may show biomarker change within weeks, whereas structural stabilization may require months and radiographic collapse-free survival requires longer follow-up. A negative MRI change at 2 weeks may be meaningless, but persistent pain and rising inflammatory markers at 6 weeks may signal failure of entry blockade. Likewise, apparent radiographic stability at 3 months may be misleading if the patient has worsening pain, edema, or endothelial injury markers. Dynamic interpretation protects against both premature escalation and delayed rescue.

## 10 Digital decision support: stage x risk x niche

Digital tools can operationalize the three-dimensional model. MRI and X-ray radiomics, deep learning segmentation, automated necrotic volume estimation, and collapse prediction algorithms are rapidly emerging. These methods can reduce observer variability, quantify lesion geometry, and integrate features that are difficult for clinicians to assess manually. However, imaging artificial intelligence should not replace biological interpretation. A large lateral lesion with low inflammatory activity and a small medial lesion with intense marrow edema and high DAMP burden may require different pathways even if a purely structural model assigns similar risk (124–131).

We propose a decision matrix defined by Stage, Risk, and Niche (**Table 6**). Stage includes ARCO/Ficat/Japanese classification, lesion volume, preserved angles, lateral extension, subchondral fracture, and collapse. Risk includes steroid dose and duration, alcohol intake, lipid profile, coagulation status, inflammatory disease activity, and bilateral or multifocal involvement. Niche includes death-program markers, inflammatory signals, vascular/endothelial markers, bone turnover balance, marrow edema, and functional trajectory. Treatment escalation is recommended when at least two dimensions are high-risk or when one dimension is extreme. De-escalation or surveillance is reasonable only when all three dimensions are stable or improving.

**Table 6:** Stage-risk-niche decision matrix for individualized ONFH management.

Dimension	Low	Intermediate	High
Stage	Small medial lesion, no edema, no subchondral fracture	Pre-collapse lesion with lateral extension or increasing edema	Large lateral lesion, crescent sign, subchondral fracture, early collapse
Risk-factor burden	Risk input removed or controlled	Partially controlled steroid, alcohol, metabolic, or coagulation burden	Persistent high-dose steroid exposure, active inflammatory disease, severe lipid/coagulation disorder
Niche activity	Improving pain/function, stable MRI, low inflammatory trajectory	Pain flares, marrow edema, rising death/inflammation markers	Progressive pain, high inflammatory/endothelial injury signals, rapid imaging progression
Suggested direction	Entry blockade and surveillance	Entry blockade plus niche reprogramming; consider decompression	Combined biological and structural reconstruction; arthroplasty if irreversible

The digital promise must be tested rigorously. Radiomics models often suffer from small samples, single-center training, heterogeneous MRI protocols, unclear label definitions, and limited prospective validation. For Frontiers in Immunology readers, the most important opportunity is to integrate imaging models with immune and niche biomarkers, allowing algorithms to predict not only collapse but also response to specific biological or immunomodulatory interventions.

A stage-risk-niche model can be implemented as a pragmatic clinical workflow. At diagnosis, the clinician records structural stage and quantitative lesion parameters; documents risk-factor burden; and obtains baseline activity measures such as marrow edema, pain trajectory, and selected blood markers. At early follow-up, the same domains are reassessed. If stage is stable but risk and niche activity remain high, biological escalation is considered. If niche activity is improving but structural stage is high-risk, reconstruction may still be necessary. If all three improve, surveillance and load management may be sufficient.

Machine-learning models should be trained to predict decisions and responses, not only collapse. A model that predicts collapse but cannot indicate whether decompression, cell therapy, scaffold reconstruction, or arthroplasty is appropriate has limited clinical utility. Conversely, a multimodal model that identifies an inflammatory transition phenotype likely to respond to niche reprogramming would directly change management. This requires prospective datasets in which interventions, biomarkers, imaging protocols, and patient-reported outcomes are harmonized from the start rather than reconstructed retrospectively.

## 11 Controversies and evidence gaps

Several controversies remain. First, the immune-editing analogy is conceptually useful but must be validated with longitudinal tissue, imaging, and blood data. Second, many cell-death pathways in ONFH are supported by preclinical or bioinformatic evidence but lack clinically actionable assays. Third, inflammation may be beneficial in early clearance and harmful in chronic persistence, so indiscriminate suppression could be counterproductive. Fourth, MSC and extracellular-vesicle therapies are heterogeneous in source, processing, dose, potency, and timing; comparing trials without quality-control standards is difficult. Fifth, structural procedures are evaluated with variable endpoints, including radiographic collapse, conversion to arthroplasty, pain, Harris Hip Score, and survival analysis, making meta-interpretation challenging.

Another unresolved issue is causality. Does inflammation cause collapse, or does microfracture cause inflammation? The loop model predicts both. Early inflammation may precede visible structural failure in some patients, whereas mechanical collapse can create secondary inflammation in others. Distinguishing these trajectories requires serial multi-omics, standardized MRI, finite-element or load-distribution analysis, and repeated functional measures. Mechanistic trials should stratify patients by activity state rather than by stage alone.

Ethical and translational questions are also important. Aggressive biological interventions in young patients must be justified by credible progression risk. Conversely, delaying structural reconstruction in a chronic escape lesion may prolong pain and reduce quality of life without preserving the hip. A transparent decision framework that defines treatment goals, escalation thresholds, and stopping rules is therefore essential.

The greatest evidence gap is the lack of repeated sampling from the human femoral head across disease phases. Most mechanistic tissue studies are cross-sectional and often use samples obtained at arthroplasty, when the lesion may already be chronic escape. Such samples are valuable but may not represent early transition biology. Blood biomarkers are easier to sample serially but may not reflect the local niche. Circulating biomarkers should therefore be interpreted as surrogate indicators rather than direct measurements of the femoral head microenvironment. Their relationship with local tissue concentrations may be influenced by tissue barriers, clearance kinetics, lesion heterogeneity, and sampling timing. Future studies should combine ethically obtained local tissue, marrow aspirates, circulating biomarkers, and imaging to connect systemic signals with femoral head biology.

A second challenge is therapeutic heterogeneity. Terms such as MSC therapy, bone marrow concentrate, exosome therapy, and biomaterial scaffold can refer to products with very different cellular composition, potency, release kinetics, and immune effects. Without minimal potency assays and reporting standards, a negative trial may reflect an ineffective product, poor timing, inappropriate patient selection, or an outcome measure that does not capture biological benefit. Immunology can help by defining mechanistic release criteria and response biomarkers for regenerative interventions. Key translational bottlenecks and potential solutions for niche-reprogramming interventions are summarized in **Table 7**.

**Table 7:** Translational bottlenecks and potential solutions for niche-reprogramming interventions.

Bottleneck	Current limitation	Potential solution
Patient selection	Stage-only decisions miss biological activity	Adopt stage-risk-niche tri-dimensional stratification
Biomarker deployment	Single timepoint assays misclassify disease phase	Use serial panels and trajectory-based interpretation
Product heterogeneity	MSC/exosome/scaffold quality varies across studies	Set potency standards and release criteria before clinical use
Delivery efficiency	Poor local retention and off-target diffusion	Develop targeted carriers and controlled-release systems
Endpoint heterogeneity	Inconsistent trial endpoints hinder comparisons	Standardize collapse, function, pain, and conversion endpoints
Data interoperability	Single-center protocols limit external validity	Build multicenter harmonized datasets with common dictionaries

## 12 Future directions

Future research should build ONFH cohorts around disease activity rather than only structural stage. A core data dictionary should include etiology, cumulative steroid exposure, alcohol and metabolic variables, coagulation markers, lesion geometry, marrow edema, DAMP/inflammatory markers, endothelial markers, bone-turnover markers, functional outcomes, and treatment timing. Such cohorts would allow the field to distinguish low-activity contained lesions from high-activity transition lesions and chronic escape lesions.

Adaptive clinical trials are particularly well suited to the proposed model. Patients could be randomized within activity-defined strata to entry blockade alone, entry blockade plus niche reprogramming, or combined biological and structural reconstruction. Interim biomarker and MRI responses could guide escalation. Primary outcomes should include both collapse-free survival and patient-centered function, while secondary outcomes should test whether death/inflammation/niche trajectories predict treatment response.

Preclinical models also need refinement. Most animal models emphasize steroid exposure or vascular disruption but do not fully reproduce human heterogeneity, bilateral disease, chronic risk-factor inputs, or load-bearing biomechanics. Models combining glucocorticoid exposure, lipid stress, endothelial injury, and controlled mechanical loading may better capture the loop. Importantly, interventions should be timed to specific phases: death-input reduction in early disease, immunovascular niche reprogramming in transition disease, and scaffold-supported reconstruction in structural-risk disease.

One practical research agenda is to create an ONFH immune-niche atlas. Single-cell and spatial approaches could map macrophage subsets, endothelial states, stromal-lineage differentiation, osteoclast-osteoblast interfaces, and death-program signatures across the necrotic core, reactive border, and distant marrow. Integrating such an atlas with MRI geometry and clinical outcomes would allow investigators to determine whether specific cellular states correspond to containment, transition, or chronic escape progression.

A second agenda is to test combination timing. Many current studies evaluate one intervention against another, but the loop model predicts that the sequence matters. Entry blockade before decompression may reduce incoming stress; niche reprogramming during decompression may improve reparative take; scaffold-supported reconstruction after niche stabilization may improve mechanical durability. Adaptive trials can compare sequences rather than isolated therapies, with early biological response used to redirect patients before collapse occurs.

## 13 Conclusion

ONFH should be viewed as more than a static consequence of interrupted blood supply. It is a dynamic osteoimmune microenvironmental disease in which multiple cell-death programs provide ongoing danger input, DAMP-PRR signaling reprograms innate immunity, the bone marrow niche loses osteogenic and angiogenic competence, and mechanical instability amplifies further injury. The proposed immune-editing-like phases - early containment, transitional coexistence, and chronic escape - provide a temporal framework for interpreting when inflammation helps clearance, when it becomes a fragile balance, and when it drives repair failure. Translating this model into entry blockade, niche reprogramming, and structural reconstruction can support individualized escalation. The next step is prospective validation using standardized stage-risk-niche measurements, dynamic biomarkers, and clinically meaningful functional endpoints.
